# Severe Visual-Auditory Impairments and Heart Failure Readmissions: Insights From the National Readmission Database

**DOI:** 10.1016/j.jacadv.2025.101895

**Published:** 2025-06-19

**Authors:** Melvin R. Echols, Ndausung Udongwo, Makoto Ikamato, Temidayo Abe, Anekwe Onwuanyi, Joseph Heaton, Jesus Almendral

**Affiliations:** a Morehouse School of Medicine, Atlanta, Georgia, USA; b American College of Cardiology, Washington, DC, USA; c Deaf Multicultural Counseling, LLC, Atlanta, Georgia, USA; d Vanderbilt University Medicine Center, Nashville, Tennessee, USA; e Hackensack Meridian School of Medicine, Neptune, New Jersey, USA

**Keywords:** 30-day readmission, accessible health care interventions, blindness, deafness, health care disparities, heart failure (HF), inpatient mortality, Kaplan-Meier survival analysis, multivariable logistic regression, neurosensory deficits (NSIs)

**What is the clinical question being addressed?**

How do visual and auditory neurosensory impairments (NSIs) impact 30-day readmission and inpatient mortality among patients hospitalized for heart failure?

**What is the main finding?**

Patients with auditory NSIs had a significantly lower readmission rate than those with or without visual NSIs, possibly indicating potential divergent outcomes not captured.

Patients with visual and auditory neurosensory impairments (NSIs) face unique challenges after hospitalization for acute heart failure (HF), potentially exacerbating adverse outcomes after discharge.^1^ Patients with these NSIs are reported to experience higher rates of hospital readmissions, longer stays, increased prevalence of psychological distress, and cardiovascular disease.^1^^,^^2^ Recent studies suggest disabilities worsen HF outcomes for patients, but the specific impact of visual and auditory NSIs in patients with HF is largely uncharacterized. Thus, we used the National Readmissions Database (NRD) to evaluate inpatient mortality and readmission rates for patients with HF and NSIs, hypothesizing that these patients have worse in-hospital mortality and 30-day readmissions following HF discharge.

This retrospective cohort study analyzed HF hospitalizations from the NRD (2016-2022) using identifiable ICD-10 codes (I50. x) for systolic, diastolic, and combined HF. Severe visual impairment (visual acuity <60/60) and severe auditory impairment, including deafness (≤80 dB), were defined by World Health Organization criteria aligned with ICD-10 codes: H54.0 to H54.3 for bilateral severe vision loss; H90.3, H91.3, and H91.8X for bilateral hearing loss ≥80 dB.^1^^,^^2^ The primary outcomes included 30-day all-cause readmission and inpatient mortality rates. Readmission and inpatient mortality were modeled using a multivariable Cox regression analysis. Covariates were selected a priori: age, zip code income quartile, insurance type, and significant comorbidities defined by the Elixhauser index. Socioeconomic insecurity was determined by using indicators with previously established ICD-10 codes.^3^ Secondary outcomes included length of hospital stay and cause of readmission. A threshold of 5% missing data was set for multiple imputations. Two variables, insurance and median household income, reported 2.49% and 1.45% missing data, respectively, so multiple imputations were not performed. The study used deidentified data from the NRD and was exempt from institutional review board approval.

Among the 1,184,502 patients evaluated in this study, 42,052 (3.56%) experienced NSIs (visual, n = 11,954; auditory, n = 28,601; combined visual and auditory, n = 1,497) (See Table 1). Patients with auditory NSIs were older and more likely to be males, whereas patients with combined visual/auditory NSIs were more likely to be female. Proportionally, there were higher 30-day readmission rates and increased length of stay (LOS) associated with patients having visual NSI, although these findings were nonsignificant (readmission 16.96%, HR: 1.06, 95% CI: 0.99-1.13; LOS β 0.19, 95% CI: 0.00-0.38). Patients with auditory NSI had a significantly lower readmission rate (13.33%, HR: 0.89, 95% CI: 0.85-0.95) and LOS compared to patients without NSI (β −0.16, 95% CI: −0.27 to 0.05). Common diagnoses for readmissions were HF exacerbation (62.4%), sepsis (9.5%), and renal complications (6.0%). Our data suggest that patients with HF and auditory or visual NSIs have divergent outcomes after acute HF discharge, requiring further study to define these differences.

**Table 1:** Baseline Demographics, Primary, and Secondary Outcomes of Patients With HF and NSIs

	All (N = 1,184,502, 100%)	Absent (n = 996,909, 96.5%)	Visual NSI (n = 11,954, 1.0%)	Auditory NSI (n = 28,601, 2.4%)	Visual and Auditory NSIs (n = 1,497, 0.1%)
Age, y (±SD)	70.72 (15.18)	70.41 (15.17)	72.97 (14.81)	81.64 (11.03)	81.72 (11.98)
Female, %	47.73	47.72	50.36	46.48	54
Elixhauser comorbidity (±SD)	4.93 (2.10)	4.92 (2.10)	5.24 (2.03)	5.20 (2.09)	5.12 (2.12)
Depression, %	11.67	11.59	13.54	13.89	17.13
Hypertension, %	58.22	58.06	69.85	59.53	65.14
Diabetes, %	38.69	38.75	50.41	31.83	35.15
Obesity, %	22.09	22.33	20	13.9	12.41
Renal failure, %	36.9	36.63	49.65	42.25	43.55
Chronic pulmonary disease, %	39.24	38.03	37.99	40.85	39.2
Socioeconomic insecurity	10.34	10.25	11.4	13.42	10.7
Inpatient mortality, n (%)	38,824 (3.28%)	37,125 (3.25%)	398 (3.33%)	1,244 (4.35%)	57 (3.78%)
# Readmission ≤30 d, n (%)	174,081 (14.70%)	168,297 (14.73%)	1,960 (16.96%)	3,645 (13.33%)	179 (11.96%)

Primary and Secondary Outcomes, HR (Reference Group: Absent NSIs)						
	LOS Coefficient (95% CI)	*P* Value	Death HR (95% CI)	*P* Value	Readmit HR (95% CI)	*P* Value
Visual NSI	0.19 (−0.00, 0.38)	0.052	0.97 (0.83, 1.12)	0.655	1.06 (0.99, 1.13)	0.12
Auditory NSI	**−0.16 (−0.27, −0.05)**	**0.005**	0.94 (0.86, 1.04)	0.223	**0.89 (0.85, 0.95)**	**<0.001**
Both NSIs	0.39 (−0.10, 0.88)	0.118	0.80 (0.54, 1.19)	0.276	0.83 (0.67, 1.03)	0.093

Overall Top 3 Readmission Diagnoses, n (%)	
Acute heart failure	64,317 (62.4%)
Sepsis	9,783 (9.5%)
Acute renal failure	6,151 (6.0%)
Other diagnoses	22,825 (22.1%)

Sub-HR for competing risk, CIs, LOS, SD. Bold values denote statistical significance of *P* < 0.01.

HF = heart failure; LOS = length of stay; NSI = neurosensory impairment.

The lower 30-day readmission rate among patients with HF and auditory NSI may imply protective measures or confer more favorable outcomes for these patients. In contrast, the potential risk of adverse HF outcomes for patients with auditory impairments, such as those with visual NSIs, is likely to present multifaceted challenges and worse outcomes that are not measured in this study. Limited provider capacity to support platforms optimizing patient-provider exchange hinders the processing and communication of health information for patients with NSIs. A recent mixed-methods analysis showed that deaf patients often experience ineffective communication due to the limited availability of interpreters, visual aids, or provider knowledge in the emergency room.^4^ These barriers may result in poor access to proper hearing or visual resources, further complicating patient risk. Inconsistent delivery of care, medication instructions, and inadequate information comprehension affect the postdischarge care of HF patients with NSIs. This study expands the literature on patients with HF outcomes associated with NSIs, emphasizing the need for continued customization of care in vulnerable HF populations.

Our study had several limitations. The retrospective design and ICD-10 classification of NSIs are subject to the inherent vulnerabilities of bias, accuracy, and completeness. The lack of pertinent information, such as race, ethnicity, zip codes, and assistive device utilization, impedes patient-level insights into social factors. The initial classification of patients with NSIs was based on ICD-10 codes aligned with complete and bilateral vision or hearing loss. Consequently, we developed criteria that enable a plausible differentiation of patients with severe NSIs, allowing for a combination of severe and total deficits for characterization based on previously published findings.^1^^,^^5^ The less stringent criteria increased the generalizability of our findings while continuing to demonstrate disparate outcomes for any severe NSI without complete vision or hearing loss.

Our study should motivate future research to explore outcomes for patients with HF and NSIs, including in-depth assessments of patient-reported outcomes. Our findings emphasize the need for targeted interventions and a better understanding of patient-reported outcomes in this population. Addressing challenges faced by patients with HF and NSIs meets part of the need to improve health care for all. These results can inspire actionable changes from clinicians, researchers, and policymakers to develop tailored interventions that mitigate disparities in vulnerable populations with HF.

## Funding support and author disclosures

The authors have reported that they have no relationships relevant to the contents of this paper to disclose.
